# Discovery, genotyping and characterization of structural variation and novel sequence at single nucleotide resolution from *de novo* genome assemblies on a population scale

**DOI:** 10.1186/s13742-015-0103-4

**Published:** 2015-12-24

**Authors:** Siyang Liu, Shujia Huang, Junhua Rao, Weijian Ye, The Genome Denmark Consortium, Anders Krogh, Jun Wang

**Affiliations:** 1 BGI-Europe, Ole Maaløes Vej 3, DK-2200 Copenhagen N, Denmark; 2 Department of Biology, University of Copenhagen, Ole Maaløes Vej 5, DK-2200 Copenhagen N, Denmark; 3 School of Bioscience and Bioengineering, South China University of Technology, Guangzhou, 510006 China

**Keywords:** *de novo* assembly, Structural variation, Novel sequence

## Abstract

**Background:**

Comprehensive recognition of genomic variation in one individual is important for understanding disease and developing personalized medication and treatment. Many tools based on DNA re-sequencing exist for identification of single nucleotide polymorphisms, small insertions and deletions (indels) as well as large deletions. However, these approaches consistently display a substantial bias against the recovery of complex structural variants and novel sequence in individual genomes and do not provide interpretation information such as the annotation of ancestral state and formation mechanism.

**Findings:**

We present a novel approach implemented in a single software package, AsmVar, to discover, genotype and characterize different forms of structural variation and novel sequence from population-scale *de novo* genome assemblies up to nucleotide resolution. Application of AsmVar to several human *de novo* genome assemblies captures a wide spectrum of structural variants and novel sequences present in the human population in high sensitivity and specificity.

**Conclusions:**

Our method provides a direct solution for investigating structural variants and novel sequences from *de novo* genome assemblies, facilitating the construction of population-scale pan-genomes. Our study also highlights the usefulness of the *de novo* assembly strategy for definition of genome structure.

**Electronic supplementary material:**

The online version of this article (doi:10.1186/s13742-015-0103-4) contains supplementary material, which is available to authorized users.

## Findings

### Background

DNA sequencing technology is advancing so fast that we are very close to being able to sequence whole human genomes routinely. This ability is likely to revolutionize diagnosis and treatment of many human diseases and generally further our understanding of human biology. An ideal DNA sequencing platform is one that provides the continuous sequences of each of the chromosomes in a genome and enables the identification of all sequence variants directly. However, owing to technical limitations, the current methods for sequencing large genomes generate reads with lengths that are typically smaller than 250 bp and with limited insert size, usually less than 20 kbp [1]. The subsequent analysis of variation in a human individual generally starts from a re-sequencing strategy, that is, a strategy based on the short-read alignment to a consensus reference sequence such as the Genome Reference Consortium human genome build 37 (GRCh37) [2, 3]. This approach has sufficient sensitivity and specificity for discovering most of the single nucleotide polymorphisms (SNPs), small insertions (typically less than one fourth of the read length) and small deletions (typically less than half of the read length) in the genome, as well as some large deletions in non-repetitive sequences (for which short-read alignment is less challenging than that for repetitive sequences) [4, 5]. However, this approach is consistently biased towards the identification of certain types of other forms of variation such as large insertions, multiple nucleotide polymorphisms (MNP), inversions, translocations and novel sequences and towards the breakpoint resolutions [3, 6].

The sequence complexity of the structural variation in individual genomes and the fact that the human genome reference sequence is imperfect introduces challenges for discovery using the re-sequencing approach [7], despite the importance of those types of variation in the definition of genome structure and disease aetiology [8]. These limitations raise interest in taking another direction in investigations of human genome variation, in which we first assemble the genome and subsequently discover the variants by analysis of the assembly-versus-assembly alignment [7]. An assembly encodes not only small variants but also large variants and is free of the artifacts present in the imperfect genome reference. The sequence-ready and nucleotide resolution characteristics of the variants obtained from the *de novo* genome assembly also enable the annotation of their ancestral state and mechanism formation. These features are known to be evolutionary and pathologically important [9, 10].

Routine use of *de novo* assembly of short reads for population-scale studies of genomic variants is complicated by the requirement of high genome sequencing coverage (≥30X), the need for sophisticated library construction strategies, intensive computer memory requirements for assembly (usually >64 GB), and the limitations of current methods for assembling highly repetitive and complex regions in the human genome [11]. However, as sequencing costs decrease and assembly programs improve, high-quality *de novo* genome assembly becomes feasible. Around thirty-seven human *de novo* genome assemblies have been released so far (see Additional file 1: Table S1). The availability of these data presents opportunities to obtain a more complete catalogue of structural variants and novel sequences than that are previously available with higher nucleotide resolution.

Before this study, we have established a framework to identify homozygous structural variants and novel sequences in two *de novo* human genome assemblies [12, 13]. As part of the Genome Denmark consortium, we also developed an improved pipeline to investigate ten *de novo* genome assemblies from Danish trios [14].

**Fig. 1:**
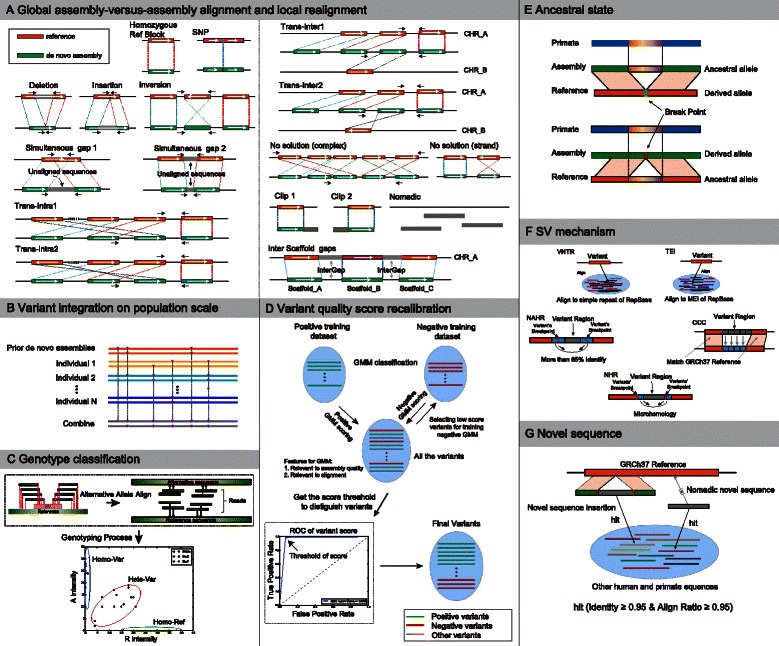
The AsmVar approach. Discovery, genotyping and characterization of structural variants and novel sequences from the *de novo* genome assemblies on a population scale. Panels **a** - **g** represent different modules. See the main text for a detailed methodological description for each module. See also Additional file 1 for the Glossary. NAHR, non-allelic homologous recombination; NHR, non-homologous recombination; TEI, transposable element insertion; VNTR, variable number of tandem repeats

In this study, we revised and extended the previous framework by integration of several novel machine-learning methods (Fig. 1, Additional file 2: Figure S1). In addition, we re-compiled the BreakSeq schemes for annotation of the ancestral state and formation mechanism of the identified structural variants and novel sequences [15]. In sum, we developed a single software package, AsmVar, to discover, genotype and characterize structural variants and novel sequences in population-scale *de novo* genome assemblies. As a proof of principle, we applied AsmVar to decipher the structural variants and novel sequence present in 10 *de novo* assemblies of trios from the Genome Denmark consortium, for which we initially develop the AsmVar approach, and other seven human *de novo* assemblies constructed using various protocols (see Additional file 1: Table S1). The results provide a direct view of the hidden genome architecture of the human population.

## Findings

### Variant discovery from assembly-versus-assembly alignment

Our approach starts with assembly-versus-assembly alignment, for which we use the LAST aligner [16] with the application of a split-alignment algorithm (Martin Frith, personal communication). In the assembly-versus-assembly alignment, we transverse each scaffold from 5’ to 3’ and record variants when mismatches, small insertions or deletions (indels) or other more complex forms of genome rearrangements are observed in one alignment block, or when breakpoints between two linear alignment blocks occur (Fig. 1a). We categorize the variations between the reference and the individual *de novo* assembly into ‘SNP’, ‘deletion’, ‘insertion’, ‘inversion’, ‘simultaneous gap’, or ‘intra- and inter-chromosomal translocation’, whereas the ones that cannot be characterized are categorized as ‘no solution’. We group the unaligned sequences in the *de novo* assembly as ‘clipped sequences’ or ‘nomadic sequences’; these are novel sequence candidates, but could also be due to contamination, assembly errors or other artifacts. The reference regions that are not covered by the *de novo* assembly are categorized as ‘inter-scaffold gaps’ or ‘intra-scaffold gaps’, and they are often associated with large repetitive sequences in the human genome or result from insufficient sequencing depth.

Around the breakpoints of the structural variants, we use an align-gap-excise alignment algorithm [17] to perform local realignment (Fig. 1a). In this process, all the variants are left-shifted and the representations of complex variants are unified, which facilitates population genetics studies of variation [18]. Subsequently, we combine all the variants from different *de novo* genome assemblies and store them in standard Variant Call Format (VCF) in accordance with the conventions of the 1000 Genomes Project (Fig. 1b) [4]. When performing this step of the approach, we recommend including the publicly available *de novo* genome assemblies from the same population (termed prior *de novo* assemblies in Fig. 1b) to increase the discovery power and provide prior information for the subsequent variant score recalibration process.

### Individual genotyping

We genotype the structural variants using a linear-constrained Gaussian mixture model with three states, AA, AR and RR, assuming that a reference allele (R) and an alternative allele (A) are segregating in the human population. The Gaussian mixture process models the density of the two-dimensional variables that record the normalized counts of reads that support the reference allele (R intensity) and the alternative allele (A intensity). Both intensities are obtained by realigning reads against the two alleles (Fig. 1c).

We constrain the centres of the three genotype states on the basis of the expected A and R intensities for each state and approximate the weight of the Gaussian mixture model by the proportion of individuals in the population with a certain genotype. We optimize the parameters in the Gaussian mixture model using an expectation-maximization (EM) algorithm with linear constraints. With the expected weight, centres and corresponding standard deviations obtained from the training process, we calculate the genotype likelihood, decide the genotype and estimate the genotype quality for each individual (see Additional file 2: Supplementary Methods for details).

In formulation, for a particular variant in the individual *i*, the genotype posterior probability of a particular genotype *j* is computed as follows:

1 $$ P\left({G}_{ij}\Big|{d}_i\right) = \frac{w_jN\left({d}_i\Big|{\mu}_j,\ {\Sigma}_j\right)}{{\displaystyle {\sum}_{j=1}^K}{w}_jN\left({d}_i\Big|{\mu}_j,\ {\Sigma}_j\right)} $$

*G*_*ij*_ represents the assumed genotype *j* for the individual *i*; *d*_*i*_ represents the two-dimension vector that composes R intensity (the count of the reads uniquely aligned to the reference allele R divided by the total depth) and A intensity (the count of the reads uniquely aligned to the alternative allele A divided by the total depth) for the individual *i*; *w*_*j*_ indicates the proportion of individuals that have genotype state *j*; *μ*_*j*_ is the expected value of mean of *d*_*i*_ given genotype state *j*; Σ_*j*_ is the expected value of standard deviation of *d*_*i*_ given genotype state *j. N*(*d*_*i*_|*μ*_*j*_, Σ_*j*_) is the probability of observing *d*_*i*_ providing the Gaussian mixture model with mean and standard deviation *μ*_*j*_ and Σ_*j*_. *K* refers to the total number of genotype states and is constantly 3 because our model only considers bi-allelic loci so far. We will release a new model that accommodates a multi-allele situation in AsmVar version 2.0.

The likelihood of observing *d*_*i*_ given a particular genotype *G*_*ij*_ is:

2 $$ P\left({d}_i\Big|{G}_{ij}\right) = {w}_jN\left({d}_i\Big|{\mu}_j,\ {\varSigma}_j\right) $$

Supposing all the individuals are unrelated to each other, the log likelihood function is constructed as follows:

3 $$ ln\ P\left(D\Big|w,\mu, \varSigma \right) = \ln \Big({\displaystyle {\displaystyle {\displaystyle {\sum}_{j=1}^K\left({\displaystyle {\displaystyle {\sum}_{i=1}^N}}\ {w}_jN\left({d}_i\Big|{\mu}_j,\ {\varSigma}_j\right)\ \right)}}} $$

*w*, *μ*, Σ are optimized using an EM algorithm with linear constraints. D refers to the set of all the observed data *d*_*i*_. The initial values for *μ* are centered around [0.001,0.001], [0.5, 0.5] and [1.0, 1.0], corresponding to the homozygous reference allele (RR), heterozygous variants (RA) and homozygous variants (AA) genotype states, respectively. These values are multiplied by a scaling factor *m* that ranges from 0.8 to 1.2 with interval 0.1 and therefore there will be five rounds of training. The best *m* is selected on the basis of the bias from a set of linear constraints and the Mendelian errors (see Additional file 2: Supplementary Methods for details). The initial value for the vector *w*, i.e. genotype frequency for three genotype states is [1/3, 1/3, 1/3].

The genotype of the individual (*G*_*ij*_) is selected as the one out of the three that has the highest posterior probability.

The Phred-scale genotype quality score (GQ_i_) is estimated by:

4 $$ G{Q}_i = -10*\  \log 10\left(1-\frac{P\left(G{T}_i\Big|d\right)}{{\displaystyle {\sum}_{j=1}^K}P\left(G{T}_i\Big|d\right)}\right) $$

### Variant quality score recalibration

Similar to the approach implemented in GATK [2], we apply a Bayesian Gaussian mixture model to the raw variant calls to assign a quality score and classify the variants as PASS and FALSE. This is a classification process guided by a positive training set, a negative training set, a set of technical features and, optimally, an independent validation set (Fig. 1d).

The positive and negative sets consist of true positive and true negative variants with additional experimental or computational evidence. We offer the users options to include their own training and validation sets. The positive sets can be the variants that are known to be polymorphic, variants independently assembled in more than one individual (double-hit events), variants that have additional computational evidence (such as the ones that are called with other software tools) or ideally variants that have been experimentally validated. The negative sets are variants known to be artifacts.

Three types of false-positive sources exist: assembly error, global alignment errors and local alignment artifacts. AsmVar captures nine metrics associated with these sources of error, including: the local assembly gap ratio; the depth of the reads that support the alternative allele; the depth of the reads that neither support the reference allele nor the alternative allele; the misalignment probability and the alignment score of the scaffolds that carry the structural variants; the local sequence identity; the position of the variants in the scaffold; and the proper aligned read ratio; and the improper aligned read ratio in the short-read versus the reference alignment (see Additional file 2: Figure S3). The users can specify all of these features or only a few selected features in the training.

We fit the quantitative measurements of a selected set of these technical features into the Gaussian mixture model and compute the log odds ratio of the likelihood that the observed variant arises from the positive training model versus the likelihood that it comes from the negative training model.

Below is the formulization of the recalibration process:

*p*_01_ and *p*_02_ are the prior probability for the variants conditioned on being positive and negative, respectively. We assign known variants with higher prior probability of being positive compared to that of the novel ones. m is the number of the cluster in the guassian mixture model ranging from 1 to the maximum number 8 by default. w indicates the size of a certain center provided m. *x* is a vector that records the distribution of the features.

5 $$ P\left(x\Big|{G}_{positive}\right) = {p}_{01}(x){\displaystyle {\sum}_{i=1}^m{w}_i}N\left(x\Big|{\mu}_i,{{\displaystyle \sum}}_i\right) $$

6 $$ P\left(X\Big|{G}_{Negative}\right) = {p}_{02}(x){\displaystyle {\sum}_{j=1}^n{w}_j}N\left(x\Big|{\mu}_j,{{\displaystyle \sum}}_j\right) $$

7 $$ {p}_{01}(x)=\left\{\begin{array}{c}\hfill 0.6,\kern0.5em x\  is\  known\  variant\hfill \\ {}\hfill \kern1.25em 0.4,\kern3.75em  Otherwise\kern2.75em \hfill \end{array}\right. $$

8 $$ {p}_{02}(x)=\left\{\begin{array}{c}\hfill 0.4,\kern0.5em x\  is\  known\  variant\hfill \\ {}\hfill \kern1.25em 0.6,\kern3.75em  Otherwise\kern2.75em \hfill \end{array}\right. $$

9 $$ Score(x)=- \lg \left(1-P\left(x\Big|{G}_{positive}\right)\right)+ lg\left(1-P\left(x\Big|{G}_{negative}\right)\right) $$

The quality score threshold is determined so as to maximize the area under the receiver operating characteristic (ROC) curve (AUC), where we keep most of the known positive variants while minimizing the inclusion of the known negative variants. It is better if the known positive and negative training variants (validation) are independent sets from the validation sets. However, when lacking such independent sets, the users can also use the option -cv in AsmVar to invoke the cross validation module, which uses the training set to assess the error rate.

Since excessive heterozygosity and homozygosity are good indicators of genotyping errors [7], we also apply the inbreeding coefficient to filter the loci with excessive heterozygosity or homozygosity (6). According to the latest investigations of artifacts in variant calling from high-coverage samples [7] and our own observations, excessive heterozygosity is relevant to the existence of large segmental duplications, whereas excessive homozygosity can derive from the assembly errors of the human genome reference or from cryptic systematic errors during data processing and variation calling.

The inbreeding coefficient (*F*) is computed as below:

10 $$ F = 1.0 - \left({N}_{\mathrm{het}}/\ \left(\ 2.0\ *p*q*N\right)\ \right) $$

Where p and q are the sample allele frequencies (only the 20 parents are considered in our study of ten Danish trios) of the reference and alternative alleles, respectively.

*N* refers to the total number of unrelated individuals in a population.

*N*_het_ refers to the total number of unrelated individuals (*N*) that are heterozygous.

By default, AsmVar removes variants with an inbreeding coefficient < -0.4 or >0.7. The threshold for inbreeding coefficient is determined based on the basis of its distribution (see Additional file 2: Figure S12), taking the GATK experience into consideration [2].

### Characterization of the ancestral state of the structural variants

After obtaining the structural variants present in the *de novo* genome assemblies, we annotate the ancestral allele state of a structural variant by comparing the identity and the aligned ratio of the reference allele and the alternative allele to the orthologous region in an outgroup genome, such as a primate genome when analyzing human sequences (Fig. 1e). By default, AsmVar uses four primate genomes (Chimpanzee panTro4, Orangutan ponAbe2, Gorilla gorGor3, Macaque rheMac3) as the outgroup genomes for comparisons. The allele that has substantially higher identity and aligned ratio to the orthologous region of the outgroup genome is identified as the ancestral allele.

We first construct the reference and the alternative alleles taking the flanking 500 bp around the variant region into account. We align both the reference and the alternative alleles to the genomes of the four primates using LAST [16] and measure the similarity using the identity and aligned ratio from the alignment. We categorize the variants as: ‘NONE’, when both the reference and the alternative alleles cannot be aligned to any of the primate genomes; ‘NA’, when both the reference and the alternative alleles can be aligned to one of the primate genomes but has less than 95 % identity and 95 % aligned ratio for all four primates; ‘Common’, when both the reference and the alternative alleles have greater than 95 % identity and aligned ratio for all four primate genomes; ‘Deletion’, when the longer allele has greater than 95 % identity and aligned ratio for any of the primate genomes and the shorter allele has less than 95 % identity and aligned ratio for any of the primate genomes; ‘Insertion’, when the longer allele has greater than 95 % identity and aligned ratio for any of the primate genomes and the shorter allele has less than 95 % identity and aligned ratio for any of the primate genomes; and ‘Conflict’, when the ‘Insertion’ and ‘Deletion’ judgment is different between different primate genomes.

Finally, we rectify the types of variation on the basis of the ancestral allele state. For example, if the assembly-versus-reference alignment suggests an insertion, but ancestral state analysis indicates that the assembly allele is the ancestral allele, we eventually annotate this variant as a deletion instead (Fig. 1e).

### Characterization of formation mechanisms of the structural variants

We characterize the formation mechanism of a variant according to the pattern of repeats in and around the variant sequence using a classification scheme similar to the BreakSeq method [19] and the 1000 Genomes Project approach [20]. Briefly, we align the variant allele sequences to RepBase using RepeatMasker [21] and perform reciprocal alignment between the left and right breakpoint sequences using BLASTn [22] (Fig. 1e; Methods). The assembly alleles that show substantial similarity with simple repeats or mobile element sequences in RepBase are annotated as variable number of tandem repeats (VNTR) or transposable element insertion (TEI), respectively. The variants that have more than 85 % identity between the two breakpoints are annotated as non-allelic homologous recombination (NAHR). Variations that contain short tracts of identical sequences around the breakpoint (micro-homology phenomena) are annotated as non-homologous rearrangements (NHR). In addition, if the full variant sequence is completely identical to the 3’ sequence of the right breakpoint, it is annotated as copy count change (CCC), which mainly derives from DNA polymerase slippage [20].

### Novel sequence

In addition to structural variants, we identify novel sequence insertions and novel sequences that are not well aligned to the consensus human genome reference but have high similarity to other human and primate genomes (identity ≥0.95 and align ratio ≥0.95) (Fig. 1f; see Additional file 2, Supplementary Methods for details). We analyse the distribution, ancestral state and mechanism of formation of all novel sequence and link the novel sequences to the closest sequences from known *de novo* assemblies.

### Scalability

AsmVar is highly efficient and currently takes only approximately 16 h to discover, genotype and characterize the structural variants and novel sequences from a *de novo* assembly using 8 CPU cores and 64 GB of memory (see Additional file 3: Table S2).

### Conventions and graphical presentation

To facilitate downstream analysis and research communication, we record the structural variants in a standard VCF [23], according to the 1000 Genomes Project convention. AsmVar also summarizes the types, size spectrum, ancestral state and formation mechanism of the structural variants and novel sequences from the investigated samples graphically in demo plots.

A complete description of the AsmVar approach is provided in Additional file 2: Supplementary Methods.

### Discovery and genotyping of structural variants from 37 human de novo genome assemblies

We show the utility of the AsmVar strategy by applying this tool to systematically investigate the structural variants and novel sequence in the currently available *de novo* assemblies of the human genome. By 31 July 2014, 37 human *de novo* assemblies are accessible to us, which include the ten Danish trios from the Genome Denmark consortium [14] and another seven *de novo* assemblies. Detailed information about the 37 *de novo* assemblies is listed in Additional file 1: Table S1. We present the results in a series of demo plots generated by the AsmVar package.

Using the AsmVar strategy, we initially identify a total of 8,609,194 raw non-SNP variants and subsequently assign genotype likelihoods, genotype and genotype quality to each individual. As a positive control set, we randomly select a subset of 626,028 double-hit exact breakpoint structural variants that are independently assembled from more than two individuals (see Additional file 2: Figure S2). We then quantify the variant quality score in the recalibration module l (see Additional file 2: Figure S3). Finally we obtain 3,176,200 structural variants from the 37 *de novo* assemblies, with lengths that range from 1 bp to 50 kbp; approximately 93 % of the positive training variants can be recovered and the false-positive rate is approximately 0.7 % (see Additional file 2: Figure S4).

**Fig. 2:**
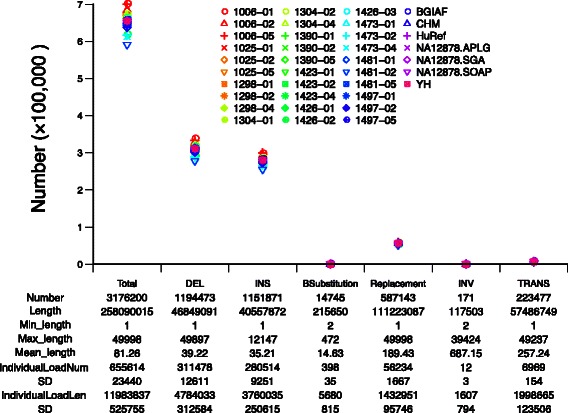
AsmVar demo plot of structural variants in the 37 human *de novo* assemblies. ‘Number’, ‘Length’, ‘Min_length’ and ‘Max_length’ indicate the total number, total length, minimum length and maximum length of structural variants that are present in the 37 human *de novo* genome assemblies. Also shown are the ‘Individual load’ and ‘standard deviation’ (‘SD’) of both the number and length of structural variants for each type of variant. The dot symbols in the plot indicates individual load

**Fig. 3:**
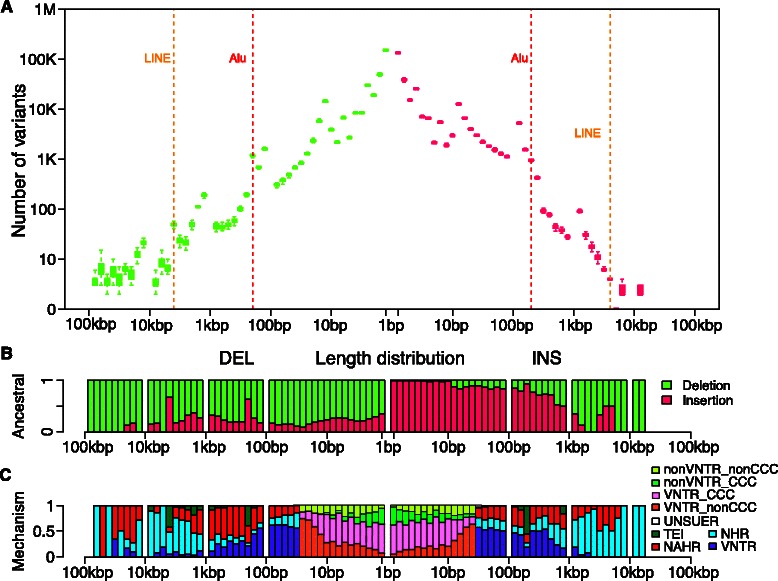
AsmVar demo plot of the ancestral state and mechanisms of origin of deletions and insertions. **a** Size spectrum of the deletions and insertions. The box plot corresponds to the quartiles of the individual load according to the size distribution. Both the *x*-axis and the *y*-axis use logarithm scales. **b** Annotation of the ancestral state of the structural variants according to the size spectrum. The *y*-axis denotes proportion. **c** Annotation of the mechanism of formation of the structural variants according to the size spectrum. The *y*-axis denotes the proportion of the variants that belong to differnet kinds of mechanisms as a function of the variation size. By default, AsmVar annotates the mechanisms of formation of structural variants <50 bp as ‘UNSURE’. NAHR, non-allelic homologous recombination; NHR, non-homologous recombination; TEI, transposable element insertion; VNTR, variable number of tandem repeats; Other abbreviations in the plot are INS, insertions and DEL, deletions

As shown in Fig. 2, our approach reveals a variety of structural variants with nucleotide resolution, which include 1,194,473 deletions, 1,151,871 insertions, 14,745 block substitutions, 587,143 length-asymmetric replacements, 171 inversions and 223,477 translocations. The variants range from 1 bp to 100 kbp, with peaks around 300 bp and 6 kbp, which correspond to transposition events that took place in the evolution of human populations (Fig. 3a). The individual load and size spectrum of the structural variants approximate those reported by the HuRef genome investigation [24], but these data have been consistently missed in genome analyses in which re-sequencing-based approaches were used. The latter mainly restricts in deletion investigations and displays substantial bias over size spectrum and resolution (see Additional file 2: Figure S5) [4, 5].

### Benchmarking the sensitivity and specificity of structural variant genotyping by AsmVar

We benchmark the AsmVar approach using both computational and experimental evidence. As 51.14 % of the structural variants identified by AsmVar (*N* = 1,624,308) are novel, that is, not present in the current dbVar database, we perform computational validation of the novel callset. By observing a random selection of 600,000 of the novel structural variants, we discover that the normalized read intensity is systematically stronger for the alternative allele than for the reference allele (see Additional file 2: Figure S6). This finding suggests that most of the novel structural variants are true polymorphisms within the human population.

We subsequently evaluate the structural variant genotyping performance of AsmVar using population metrics including family relatedness and the Mendelian error rate. Those metrics are computed using the PLINK software [25]. The probability of identity by descent being equal to 1 (IBD1) for the parent-offspring genomes varies from 0.02 to 0.14 for deletions and 0.10 to 0.19 for insertions, whereas the probability of pairwise IBD0 for unrelated individuals approximates zero (see Additional file 2: Figure S7). The Mendelian error rate ranges from 0.01 to 0.21 for deletions and 0.03 to 0.10 for insertions (see Additional file 2: Figure S8). Based on these metrics, we estimate that the genotyping error for AsmVar calls is approximately 2 % to 20 %. Although the performance of AsmVar for structural variant genotyping is not as good as that for GATK SNP identification, the genotyping accuracy of AsmVar substantially exceeds that of the most widely used software for structural variation genotyping, GenomeStrip [6], which was the structural variation caller and genotyper adopted in the 1000 Genomes Project (see Additional file 2: Figure S7 and Figure S8).

Furthermore, we benchmark the performance of AsmVar using two datasets for which experimental evidence exists. First, as NA12878, which is included in our study, is a well-studied individual genome, we benchmark the sensitivity of AsmVar by comparing the NA12878 AsmVar non-reference genotype calls to the 21415 dbVar structural variation records for this individual [5]. These structural variants include 18,108 deletions, 294 insertions, 491 duplications and 39 inversions that are >50 bp and were validated by different experimental approaches. Also, there were 2050 deletions, 152 insertions, 244 duplications and 37 inversions that failed experimental validation.

Among the validated structural variants, 3738 are missed by AsmVar without enrichment of a certain size spectrum (see Additional file 4: Table S3). Therefore, the overall false-negative rate of AsmVar is approximately 20.1 %. Manual investigation into these missing calls suggests three main reasons for false-negative calls: 1) assembly gaps due to insufficient coverage; 2) assembly gaps derived from long repetitive sequences; and 3) assembly errors probably result from underlying complex genomic sequences.

AsmVar calls none of the 2483 variants from the NA12878 dbVar dataset that failed validation. However, as the true number of variants present in NA12878 is not available at the moment based on our observations of the Illumina Platinum Genomes and Genome In A Bottle datasets [18], we are not able to unbiasedly assess the false-positive rate of AsmVar using the NA12878 public data. In addition, as genotype information about structural variants in the NA12878 dbVar records is not available, we cannot benchmark the genotyping accuracy of AsmVar using the dbVar information.

To further assess the specificity of AsmVar in structural variation discovery, we randomly select one Danish trio from the Genome Denmark consortium and validate 272 novel structural variants with a range of different sizes (≥50 bp) and formation mechanisms using the Sanger sequencing technology [14]. We successfully assay 68 structural variants, and from this analysis we estimate that the overall false-discovery rate of AsmVar for structural variants is 7.4 % (5/68, 95 % confidence interval = 0.03-0. 16) (see Additional file 5: Table S4). For the remaining 204 loci, 158 are not successfully assayed because of failure in primer design and 46 are not successfully assayed because of other experimental problems, such as the failure of the PCR or sequencing.

The validation of structural variation remains a challenge. The experimental failure rate is high, probably because most of the structural variants occur in repetitive sequences of DNA. We therefore include in the AsmVar package an extension script to plot out the proper and the improper read coverage at and around the loci in which structural variation was identified (see Additional file 2: Supplementary Methods, for definition of proper and improper reads; see also Additional file 2: Figure S9). Manual inspection indicates that the false-positive rates for the two categories of failure attempts are 6.5 % and 8.2 %, respectively. Owing to the limited number of validation loci available for each size band or for each type of formation mechanism, we cannot correlate the false-discovery rate with the size spectrum and the formation mechanism of the variants with high confidence.

### The ancestral state of the structural variants

One characteristic of the variants in AsmVar is that their sequences are available, which is the precondition to define the ancestral state of a variant. To obtain insight into the evolutionary origin of the structural variants obtained from the 37 human *de novo* assemblies that were included in this study, we apply AsmVar to analyze the ancestral state of the variants according to the size spectrum. We summarize the AsmVar results using the demo plot functionality (Fig. 3b). Owing to the lower quality of some of the primate genomes when compared with that of the human *de novo* assemblies, we cannot characterize the ancestral state of 51.2 % of the variants. By comparing the human datasets to the outgroup genomes, we discover that 9 % of the insertions in the *de novo* assemblies show higher similarity to the outgroup genomes than to the human reference genome and are indeed evolutionally deletion events in the first beginning. This observation also highlights the incompleteness of the consensus human genome reference (Fig. 3b). Conversely, we discover that 28 % of the classified deletions are instead insertion events. Consistent with the molecular level understanding, the deletions that have arisen owing to TEI mechanisms tend to be insertions in the historical course (Fig. 3b). Our approach reveals similar patterns of distribution of ancestral states among structural variants than those reported in previous population-scale investigations in which a set of large deletions and a very limited number of tandem duplications were analyzed [5].

### The formation mechanism of the structural variants

Nucleotide resolution of the structural variants identified using AsmVar enables the characterization of their mechanisms of formation. We classify the mechanisms of formation of the structural variants into VNTR, TEI, NAHR, NHR and CCC, i.e. copy number changes derived from a DNA polymerase slippage process across the size spectrum (Fig. 3c). Our approach demonstrates a symmetric view of mechanisms distribution corresponding to our molecular level understandings. Most of the 1–10 bp insertions and deletions have exact copy number changes that are relevant to DNA polymerase slippage. The 300 bp and the 6 kbp variants are enriched in TEI and the larger variations (>1,000 bp) arise from NAHR and NHR, whereas the smaller ones are enriched in VNTR [15]. Most of the TEI-derived deletions indeed have insertions as the ancestral state. These observations follow our biological intuition, which indirectly proves the robustness of our approach.

### Novel sequence

**Fig. 4:**
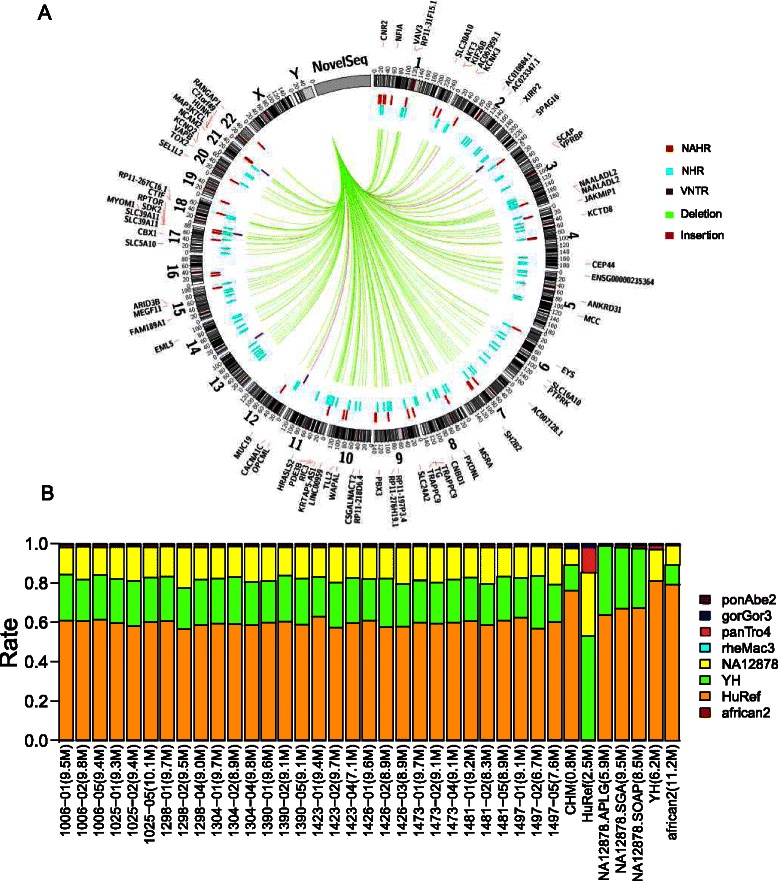
AsmVar demo plot of novel sequence identified in this study. **a** Distribution of the novel sequence insertions (> = 100 bp) over the different human chromosomes, with their mechanisms of origin (NAHR, NHR, VNTR) and their ancestral states (deletion and insertion) shown from outside to inside. **b** Total length (*x*-axis) and distribution of the closest relativeness of the nomadic novel sequence. NAHR, non-allelic homologous recombination; NHR, non-homologous recombination; TEI, transposable element insertion; VNTR, variable number of tandem repeats

In addition to structural variants, we identify 9 million base pairs of novel sequences (>100 bp), on average, per individual that are not present in the human genome reference sequence, as shown in the AsmVar demo plot (Fig. 4).

We divide the novel sequences into novel sequence insertions and nomadic novel sequences (Fig. 1g). We first investigate the ancestral state, the formation mechanism and chromosomal distribution of the novel sequence insertions. 90 % of the novel inserted sequences show higher similarity to the outgroup primate genomes compared to the human reference genome. Therefore, we observe a higher number of deletions than insertions in the ancestral state analysis, which correspond to NHR and NAHR molecular mechanisms of origin [19] (Fig. 4a). The novel sequence insertions are distributed across the whole human genome, affecting the structure of 71 genes. We randomly select 18 large novel sequence insertions (≥1 kbp) and apply quantitative PCR (qPCR) to validate their existence. Manual observation of the electrophoretic band validates all of these insertions (see Additional file 6: Table S5). However, AsmVar predicts the insertion length incorrectly for one locus.

We subsequently learn about the un-localized novel sequences identified by AsmVar by linking each of the sequences of one individual to their closest neighbour (Fig. 4b). We notice that CHM assembly contains a very limited number of novel sequences and confirm that this assembly is a reference-guided *de novo* assembly. This finding also highlights a bias of re-sequencing-based approaches for investigation of genome variation. Except for CHM genome assembly, we observe that the proportion of nomadic sequences decreases as the quality of the *de novo* assembly increases. We reason that a high-quality *de novo* assembly contains novel sequences that cannot be captured by *de novo* assemblies with lower quality. When investigating the closest relatives of the novel sequences, we observe a consistent ranking of proportion from HuRef to YH and NA12878, which corresponds to the quality of these *de novo* assemblies. These observations indicate that obtaining a comprehensive profile of the variations present in a human genome relies on high-quality *de novo* assemblies (Fig. 4b).

## Conclusions

We have presented a novel and efficient approach for discovering, genotyping and characterizing the structural variants and novel sequence from population-wide *de novo* genome assemblies.

We have implemented several state-of-the-art bioinformatics algorithms and techniques in the software. We applied a sophisticated genome-versus-genome comparison strategy that efficiently integrates the split-alignment algorithm from LAST [16] and the align-gap-excise algorithm from AGE [17], and subsequently implement an efficient method to initially identify various forms of structural variation and novel sequence from the assembly-versus-assembly alignment. We implemented a statistical approach to genotype the structural variants based on the information from reads. By using a machine-learning approach to distinguish the true variants from technical artifacts, we recover the structural variants and novel sequence from the *de novo* assemblies with good sensitivity and specificity. In addition, we include in the AsmVar package systematic supportive functionality for biological interpretations of the data, such as annotation of ancestral state and mechanism of origin of the structural variants and novel sequences, which is of great interest to human population genetics and clinical applications.

We applied the AsmVar to the 37 human *de novo* genome assemblies used in this analysis and revealed a wide spectrum of human genomic variation present in the human population, including large deletions but also insertions and other complex forms of structural variation, as well as novel sequences, which are usually missed in human population studies at present. The sequence-ready and nucleotide resolution characteristics of the AsmVar calls also enable downstream fine-scale investigations into the ancestral state and formation mechanism of structural variants and novel sequences. These novel insights reflect the limitations of re-sequencing strategies and underscore the promise of the *de novo* assembly-based analysis strategy.

We are considering extending and improving AsmVar. The current genotyping approach is practical but requires alignment of the short reads towards both the reference and the *de novo* assemblies and thus is laborious. Furthermore, the current approach does not accommodate multi-allelic loci very well. To improve the efficiency of this process and to improve the integration of population information, we are developing a probabilistic reference-alignment-free kmer-based approach that can directly obtain the allele intensities from the raw reads, which therefore reduces effort and will offer a solution to genotyping novel sequences (Lasse et al., manuscript in preparation).

Finally, we note that the quality of the *de novo* genome assembly is an important limiting factor for AsmVar analysis. The difficulties in assembling complex genomic regions such as HLA, KIR and long repeats display inferior performance [11]. Nonetheless, the current version of AsmVar offers high-quality calls and interpretations of structural variants and novel sequence present in the human populations from analysis of *de novo* genome assemblies. As sequencing and computational costs decrease and experimental technologies and *de novo* assembly algorithms evolve, more and more high-quality *de novo* assemblies from a population will become available. These assemblies are essential resources and great opportunities for us to carry out in-depth investigations into structural variation and novel sequence in the population and to construct a population-wide pan-genome. We hope the future developments and improvements of AsmVar will contribute to the comprehensive profile of the structural variants and novel sequences in different populations.

## Methods

### Sanger sequencing validation of the structural variants (≥50 bp)

We picked one trio (trio 1298) from the Genome Denmark consortium and validated a randomly selected set of variants present in the trio genomes using Sanger sequencing. The selected variants included 272 novel structural variants with different sizes and mechanisms of origin. We designed primers using an in-house pipeline integrating Primer3 and Primer-Blast. We sequenced the successfully amplified PCR amplicons with the Sanger AB3730xI DNA Analyzer. We subsequently analyzed the chromatograms using PolyPhred 6.1849 to genotype SNPs and small indels. Hereafter, all calls were manually inspected using Chromas 2.11.

### qPCR validation of the novel sequence insertions (≥1 kbp)

We designed primers over the flanking regions of the novel sequences. For a true novel sequence, we expected to observe two bands with size differences of more than 1 kbp if the selected individual was heterozygous for the variant or two bands with a size that is greater than the reference length if the selected individual was homozygous for the variant. The size of the band was estimated by manual inspection of the electrophoretogram. To provide higher resolution for the band size, we applied the predicted product length of the reference allele and the alternative allele by *in silico* PCR using Primer-BLAST.

### Evaluation of false-negative and false-positive rates of structural variant discovery in NA12878

We downloaded the structural variation list from the 1000 Genomes Project pilot project and extracted the 18,932 structural variants that were validated in NA12878. We defined false-negative calls as the structural variants that are present in the NA12878 dbVar calls dataset but for which AsmVar did not emit a non-reference genotype call that has more than 50 % reciprocal overlap for the variants from the NA12878 individual.

### Availability and requirements

Project name: AsmVar

Project homepages: https://github.com/bioinformatics-centre/AsmVar

Operating system(s): Unix, Linux, Mac OS X

Programming language: C++, Python, Perl

Other requirements: C++ 4.7.0 or higher, Python 2.7.0 or higher, Perl 5.10.1 or higher

License: GNU GPL

### Availability of supporting data

The source code for AsmVar is available at https://github.com/bioinformatics-centre/AsmVar. Example data and snapshots of the code are also available in the *GigaScience* GigaDB database [26].

The NA12878 dbVar variants are available via [27].

The assembly sequences of the four primates used in the ancestral state annotation are downloaded from UCSC [28–31].

## Additional files

Additional file 1: Table S1. Information of the 37 Human genome de novo assemblies that are used in this analysis. (XLSX 11 kb) Additional file 2: **Glossary, Supplementary Methods and Supplementary Figures S1-S12.** (DOCX 2871 kb) Additional file 3: Table S2. Memory and CPU time of AsmVar for the 37 de novo assembly investigation. (XLSX 9 kb) Additional file 4: Table S3. Assessment of AsmVar false negative rate by comparison of NA12878 validated structural variants. (XLSX 10 kb) Additional file 5: Table S4. False positive rate of AsmVar evaluated by Sanger sequencing validation. (XLSX 303 kb) Additional file 6: Table S5. qPCR to validate 18 novel sequences > 1 kbp in trio 1298. (XLSX 19 kb)

## Abbreviations

BP: base pair
CCC: Copy count change
IBD: Identity by descent
INDEL: small insertion or deletion
NAHR: Non-allelic homologous recombination
NHR: Non-homologous rearrangements
SNP: Single nucleotide polymorphism
TEI: Transposable element insertion
VCF: Variant call format
VNTR: Variable number of tandem repeats
