# Supplementary material for: Discovery, genotyping and characterization of structural variation and novel sequence at single nucleotide resolution from de novo genome assemblies on a population scale
Source: Gigascience. 2015 Dec 24;4:64. doi: 10.1186/s13742-015-0103-4 (PMC4690232; doi:10.1186/s13742-015-0103-4)
Supplement: Additional file 2: — Glossary, Supplementary Methods and Supplementary Figures S1-S12. (DOCX 2871 kb) [file 13742_2015_103_MOESM2_ESM.docx]

# Supplementary material

# Discovery, genotype and characterization of structural variants and novel sequence at single nucleotide resolution from *de novo* genome assemblies on a population scale

Siyang Liu^1,2＊^, Shujia Huang^1, 3＊^, Junhua Rao^1＊^, Weijian Ye^1＊^, GenomeDK consortium^II^, Anders Krogh^2$^ & Jun Wang^1,2$^

^1^BGI-Europe, Ole Maaløes Vej 3, DK-2200 Copenhagen N, Denmark

^2^Department of Biology, University of Copenhagen, Copenhagen, Denmark

^3^School of Bioscience and Bioengineering, South China University of Technology, Guangzhou, China

^II^ A list of members and affiliations is provided in the supplementary material

^＊^These authors contributed equally to this work

^$^ Correspondence should be addressed to A.K. (krogh@binf.ku.dk) and J.W. ([wangj@genomics.cn](mailto:wangj@genomics.cn))

Content

[Supplementary material 1](#_Toc428348863)

[Discovery, genotype and characterization of structural variants and novel sequence at single nucleotide resolution from de novo assemblies on a population scale 1](#_Toc428348864)

[1. Glossary 2](#_Toc428348865)

[2. Supplementary Notes 3](#_Toc428348866)

[Module a: Alignment and variant discovery 3](#_Toc428348867)

[Module b. Variant integration on a population scale 5](#_Toc428348868)

[Module c. Individual genotyping 5](#_Toc428348869)

[Module d. Variant quality score recalibration 9](#_Toc428348870)

[Module e. Annotation of Ancestral State 11](#_Toc428348871)

[Module f. Annotation of Mechanism 12](#_Toc428348872)

[Module g. Novel sequences analysis 13](#_Toc428348873)

[Evaluation of the false negative rate of AsmVar 13](#_Toc428348874)

[Sanger sequencing validation of the novel structural variants 14](#_Toc428348875)

[3. Supplementary figures 14](#_Toc428348876)

[4. Supplementary tables 23](#_Toc428348877)

[5. Reference 24](#_Toc428348878)

## Glossary

**Alignment block**: a continuous alignment between two sequences that may contain mismatches or INDEL

**Block substitution**: complex variation that presents same length between reference sequence and assembly sequence in the alignment breakpoints

**Clip**: sequences present at the edges of the de novo assemblies that cannot be aligned to the human genome reference

**Deletion**: deleted sequence in the de novo assembly causing a breakpoint in the assembly-vs-reference alignment

**Double-hit structural variants**: the structural variants that are independently assembled in at least two de novo assemblies

**Homozygous Ref Block**: alignment block that display misalignment probability less than 0.01 contains no gaps and display average identity greater than 99.9%.

**Insertion**: inserted sequence in the de novo assembly causing a breakpoint in the assembly-vs-reference alignment

**Intra-scaffold gap**: sequences present in the reference where only partial of which has been reconstructed in the de novo assembly

**Inter-scaffold gap**: sequences present in the reference but have not been reconstructed in the de novo assembly probably due to existence of large repetitive sequence or lack of coverage

**Inversion**: inverted sequence in the de novo assembly compared with reference

**Nomadic scaffolds**: entire scaffolds that cannot be aligned to the human genome reference

**No solution**: difference observed in the assembly-vs-assembly comparison but the variant types cannot be classified into INDEL, Deletion, Insertion, multiple nucleotide polymorphism (MNP), Inversion or translocation

**Novel sequence**: sequences that are present in the de novo assembly but have not been constructed in the public human genome reference

**Replacement**: The same as Simultaneous gap

**Simultaneous gap**: complex variation that presents different length between reference sequence and assembly sequence in the alignment breakpoints. These are also called as MNP, i.e. multiple nucleotide polymorphisms.

**Translocation**: translocated sequence in the de novo assembly compared with reference

## Supplementary Methods

The structure of the following text is based on Figure1 and FigureS1. Each module of AsmVar may contain a few steps.

### Module a: Alignment and variant discovery

#### Step1. Global assembly-vs-assembly alignment using LAST

In this step, we make pair-wise comparisons between individual de novo assembly and the human genome reference using LAST (Kiełbasa, Wan, & Sato, 2011, <http://www.cbrc.jp/~martin/talks/split-align2.pdf> ).

LAST implements split alignment algorithm, provides misalignment probability and is developed with the aim to facilitate structural variation identification from pair-wise alignments between the two sequences.

The output format is in MAF (Multiple alignment format) which can be subsequently converted into BAM format to facilitate IGV visualization.

The recommended alignment protocol between two human genome assemblies is as follows:

| Step | Commands and Parameters | Description |
| --- | --- | --- |
| lastal and last-split | lastal -e25 -v -q3 -j4 human_g1k_v37_decoy.fasta.lastdb $asm.fa \| last-split -s35 –v >$alignment.maf | Assembly-vs-reference alignment |
| maf-convert | maf-convert.py –f human_g1k_v37_decoy.repeatmask.fasta.dict sam $alignment.maf \|samtools view –bS –o $alignment.bam | Convert maf alignment format to bam format for visualization of assembly-vs-reference alignment |

##### Application notes:

1. There are other popular genome comparison tools such as LASTZ, MUMMER and BWA MEM, etc. They all adopt the seed-chain-extension protocol first put forward by BLASTZ. The following table records a few of the key characteristics for the choice.

|  | Data structure | Scalability | Split-alignment | Mapping quality estimation |
| --- | --- | --- | --- | --- |
| LAST | Suffix array | Possible for human genome vs human genome comparison | Yes | Yes |
| LASTZ | Hash table | Possible for human genome vs human genome comparison | No | No |
| MUMMER | Hash table | Possible for human genome vs human genome comparison | No | No |
| BWA MEM | Suffix array | Possible for query length less than 1Mbp before June, 2014 | Yes | Yes |

#### Step2. AGE realignment

We implement the align-gap-excise alignment algorithm ^2^ to locally realign the de novo assembly towards the reference around the breakpoints of the variants in an efficient way.

The aims of the AGE module are to

1) generate the exact breakpoints of the variants in the cases where repeat sequences occur around the SV breakpoints, blurring the true alignment

2) left-shift the variants where local alignment ambiguity exist due to existence of tandem repeat

3) unify the different representations of the same variant in complex region

4) ensure 1-based coordinates for accuracy in the “Genotyping module”

5) remove false positive calls where excessive substitutions or indels exist in the alignment of the flanking regions

#### Step3. Identification of anomalous alignment events

In the assembly-vs-assembly alignment, each scaffold from the de novo assembly is transversed from 5' to 3' and variants are emitted when mismatches, gaps (insertions or deletions) or alignment breakpoint occur. We characterize the difference between the reference and the individual assembly into “SNPs”, "Deletion", "Insertion", "Simultaneous gaps", "Inversion", "Translocation" while the ones that cannot be characterized are defined as "No solution". We term the unaligned sequences in the de novo assembly as "Clipped sequences" or "Nomadic" and the reference region that is not covered by the de novo assembly as “Inter-scaffold gaps”. Notice that besides the true variants, the difference between the individual assembly and the reference can be technical artifacts derived from misassembly and misalignment which will be treated in the following AGE and structural variation quality score recalibration modules. Also, local realignments around the variant breakpoints are required to facilitate population-scale analysis.

### Module b. Variant integration on a population scale

If there are multiple individuals, the vcf from each individual de novo assemblies are combined using the CombineVariant module in GATK ^3^ and the multiallelic records are broken into multiple records usings vcfbreakmulti in vcflib ( https://github.com/ekg/

vcflib ).

### Module c. Individual genotyping

***Step1. Alignment of short reads towards reference and the de novo assembly***

All reads are aligned to both the reference and the assgbly using bwa-mem, respectively. For each base in both the reference and the assembly, reads with mapping quality equal to or greater than 30 (indicating that the alignment error of the read is equal to or less than 0.001) covering this base are taken into account and are categorized into two types of aligned reads- proper aligned reads, improper aligned reads, reflecting evidence of the reference allele and the alternative allele, respectively (see the following Table1 for definition of the two types of aligned reads).

Table1. Characterization of the read alignments with mapping quality >=30

| Type of alignment | Description | Categories |
| --- | --- | --- |
| TOTAL_COV | Total read coverage | Proper aligned + Improper aligned |
| PROPER_PAIR_COV | Reads that are  1) aligned in pairs in the same chromosome  2) have correct fragment orientations  3) expected insert size  4) have alignment score greater than 90.  Contain capital P in the flag of bam file and the AS > 90 and therefore contains no gaps and no clips | Proper aligned |
| CLIP_AND_SA_COV | The previous base or the latter base of the current base is clipped (S in cigar) | Improper aligned |
| SINGLE_END_COV | Single end alignment  Reads that are aligned to different chromosomes | Improper aligned |
| LOW_ALIGN_SCORE_PROPER_PAIR | Proper aligned reads with alignment score <= 95 | Improper aligned |
| CROSS_READ_COV | Proper aligned reads containing gaps for the current bases | Improper aligned |
| WRONG_ORIETATION_COV | 1) aligned in pairs in the same chromosome  2) erroneous fragment orientations (fq1 and fq2 same orientation or outer alignment) | Improper aligned |
| BAD_INSERT_COV | 1) doesn't contain capital P in bwa bam which takes the insert size into considerations  2) aligned in pairs in the same chromosome  3) correct fragment orientations | Improper aligned |

# For bwa mem, the penalties for mismatch is 4, for gap open is 6 and for clip is 5.

#### Step2. Alternative allele align

Due to the intrincit extensive homologous sequence around the breakpoints of the structural variants, we observe that more than 80% of the variants contain reads at the breakpoints that are both aligned to the reference allele and the alternative allele perfectly with 100% identity and 100% aligned length in the HuRef simulation data (Data not shown), consistent with previous observation ^3^ and is the known culprit for abnomalous inbreeding coeffcient observation of indels genotypes in the population. This characteristics causes confusion in genotyping of the structural variants since even for homozygous variant allele, we will systematically observe extensive number of reads supporting the reference allele. Therefore, we divide all the reads aligned with mapping quality >=30 at and around the 5’ breakpoints to four categoreis: 1. Reads perfectly and uniquely support the reference allele 2. Reads perfectly and uniquely support the alternative allele 3. Reads perfectly support both the reference and the alternative allele 4. Reads that are both imperfectly aligned to the reference and alternative allele. We only use type1 and type2 reads to do genotyping in Module4. For multi-allelic loci, the above 4 types of reads are obtained based on the allele that belongs to that specific individual.

#### Step3. GMM Model for Genotyping

For each variant, after obtaining the reads that unambiguously support the reference allele (R) and that unambiguously support the alternative allele (A), we obtain the genotype likelihoods for each individual by fitting a two dimentional linear constraint Gaussian mixture model.

Below is the model building procedure.

##### Definitions

*N* : number of individuals in the population

*j* : genotype state where number indicates the number of the selected alternative allele (0: homozygous reference; 1: heterozygous variant; 2: homozygous variant)

*i* : individual

*K*: number of genotype states in the population of the investigated variant. K={1, 2,3}

*G_i_* : the genotype of individual i

*w_j_* : proportion of individuals that have genotype state j

*d_i_*: the data that we use as the feature A/(A+R) and R/(A+R) which represents normalized evidentiary read count for either the reference allele or the variant allele in individual i.

*µ_j_*: expected *d_i_* given genotype state j

m: scaling factor of *µ* . m∈[0.8, 1.2]

σ*j*: expected standarsd deviation of *d_i_* given genotype state j

##### The Gaussian mixture model

For a particular variant in the individual i, the genotype posterior probability of j is calculated as follows,

$P\left( G_{ij} | d \right)= \frac{w_{j}N(d_{i}|\mu_{j}, \Sigma_{j})}{\sum_{j=1}^{K} w_{j}N(d_{i}|\mu_{j}, \Sigma_{j})}$ (1)

The likelihood of observing $d_{i}$ given a particular genotype is

$P\left( d_{i} \right|G_{ij})= w_{j}N\left( d_{i} | \mu_{j}, \Sigma_{j} \right)$ (2)

Because all the parents (N=20) is unrelated with each other, the log likelihood function is constructed as follows,

$$\ln P\left( D | w,\mu,\Sigma\right)= \sum_{i=1}^{N} ln\left( \sum_{j=1}^{K} w_{j}N\left( d_{i} | \mu_{j}, \Sigma_{j} \right) \right)$$

$w,\mu,\sigma$ are optimized using an expectation-maximization algorithm with linear constraints.

The best K and m are selected based on the bias from the linear constraints and mendelian errors.

##### Expectation and Maximization ( EM ) for a certain K and m

###### Initialization

w = 1/K

µ = m * ([0.001,0.001], [0.5, 0.5], [1.0, 1.0]), m=np.linspace(0.8,1.2,10)

$$\sigma^{2}=\left( \left[ \begin{matrix} 0.002 & 0 \\ 0 & 0.002 \end{matrix} \right],\left[ \begin{matrix} 0.002 & 0 \\ 0 & 0.002 \end{matrix} \right],\left[ \begin{matrix} 0.002 & 0 \\ 0 & 0.002 \end{matrix} \right] \right)$$

###### Expectation and maximization

At most 50 iterations are performed until convergence of log likelihood (Ɛ < 10^-3^) in the Expectation step. w, µ and $\sigma$ are updated in the Maximization step. The raw likelihood and the posterior probability of the genotype of each individual is determined using formula (1) and (2), respectively.

###### Linear constraints

The final µ' returned by EM must not be biased from the original centers [0.001,0.001], [0.5, 0.5], [1.0, 1.0] by [0.8 - 1.2]. Otherwise, a new scaling factor m will be selected for a new round of EM. If no scaling factors meet the requirements, a new K, i.e. the current K minus 1 will be chosen for new rounds of EM. The linear constraints are important to avoid obtaining a local maxima that does not obey human intuition.

##### Selection of m and K

Bias from the linear constraints b = ( µ'_HomoVar_ - µ_HomoVar_ ) + abs (µ'_HeteroVar_ - µ_HeteroVar_) + (µ_HomoRef_ - µ'_HomoRef_) given that K=3. The smaller the bias, the more confident we are that the genotypes are correct. The final scaling factor m and the number of components K are chosen to minimize the bias. We have also tested the involvement of Mendelian errors in the model selection and initially prefer the final scaling factor m and the number of components K are chosen to minimize the bias and the mendelian errors. However, the Mendelian error consideration is deleted because we notice that smaller K always results in smaller mendelian errors. Using the initial 10 trio, the proportion of variants with K less than 3 is around 10%.

##### Assignments of GT_i_ and GQ_i_ for individual i

The genotype of the individual (GT_i_) is selected as the one with the highest posterior probability.

Phred-scale genotype quality (GQ_i_) is estimated by

${GQ}_{i}= -10* log10(1-\frac{P\left( {GT}_{i} | d \right)}{\sum_{j=1}^{K} P\left( {GT}_{i} | d \right)})$

For K<3, we assign 65535 as the likelihood for the rest of the genotype that cannot be obtained in the maximization step.

### Module d. Variant quality score recalibration

Artifacts and real events tend to be represented as different clusters using a set of features and the clusters are generally gaussianly distributed ^3^. To provide statistical measurements of how confident we are about the observed polymorphism, we use a supervised gaussian mixture module to assign quality scores for each varaints based on a **positive training set**, **a negative training set** and **the selected technical features of those variants**. Ideally, **the positive training set** should be a sufficient number of variations that have been experimentally validated. However, when such as dataset are not available, we can also use those variants that are already known such as strucutral variations recorded in dbSNP or dbVar. They can also be the variations indepently assembled for more than one individual which we call as the “double-hit events” . **The negative training dataset** can be those variants that fail experimental validation. Nonetheless, if such a dataset is not available, AsmVar will automatically compose the negative training set from the variants that display the lowest LOD (logarithm of the odds) value under the trained gaussian mixture model using the positive training set. Eventually, for each variant, we measure the Phred-scale variant quality using log odds ratio of the variant arising from the “good site model” versus that from the “bad site model”.

**Building the Gaussian model of the “good sites” using the selected features**

We estimate the likelihood that the variant derives from the positive gaussian mixture model (1) . m is the number of the cluster in the guassian mixture model ranging from 1 to the maximum number 8 by default. w indicates the size of a certain center provided m. $x$ is a vector that records the distribution of the features. The model paramters are obtained using an EM algorithm. $p_{0}$ is the prior probability of the variants and we assign the variants with higher prior probability if it’s known among the population, otherwise, we assign them with lower prior probability (2). We assign known variants with lower prior probability compared to the novel ones.

$P\left( x | G_{positive} \right)= p_{0}(x)\sum_{i=1}^{m} w_{i}N(x|\mu_{i},\sum_{i})$ (1)

$p_{0}\left( x \right)=\left\{ \begin{aligned} 0.6, x is known variant \\ 0.4, Otherwise \end{aligned} \right.$ (2)

**Obtaining the bad sites and build Gaussian model of the “bad sites”**

We assign the likelihood of being true for the additional loci in the vcf file based on the model obtained from the above training process. We categorize those variants that display the lowest likelihood of being true as the “bad sites” (3). We automatically decide the quality threshold as less than 1% of the training positive sites (good sites) become bad sites. An additional Gaussian model is established using those bad sites using similar approach as indicated above. We assign known variants with higher prior probability compared to the novel ones.

$P\left( X | G_{Negative} \right)= p_{0}(x)\sum_{j=1}^{n} w_{j}N(x|\mu_{j},\sum_{j})$ (3)

$p_{0}\left( x \right)=\left\{ \begin{aligned} 0.4, x is known variant \\ 0.6, Otherwise \end{aligned} \right.$ (4)

**Assigning variant quality score for the full dataset and calculation of the key feature that results in bad variant quality score**

For each variant, the variant quality score (logarithm of the odds, lod score) is calculated as log (good sites model likelihood) – log (bad sites model likelihood). For each variant, the lod score is also calculated for different features independently using the mean and standard deviation of the selected Gaussian model for the “good sites” and “bad sites” and the feature that display the lowest variant quality score is identified as the key artificial technical feature (4).

$Score\left( x \right)=-\lg\left( 1-P\left( x | G_{positive} \right) \right)+lg(1-P(x|G_{negative}))$ (5)

**Determination of variant quality score based on ROC**

We decide the variant quality score threshold to maximize the area under the ROC curve (AUC). It’s common to observe from the population variant list that variants may overlap with each other due to the existence of local repetitive sequence. In those cases, AsmVar chooses the most probable allele as the one with the highest variant quality score until no overlapping is observed.

**Post-filtration**

We observe that the calls from the above process may display excessive heterozygosity or excessive homozygosity. The former arises from misaligning reads to the paralogous loci while the latter may arise from assembly errors from the human genome reference. By default, we filter the variants with inbreeding coefficient less than -0.4. or greater than 0.7.

### Module e. Annotation of Ancestral State

The age of the polymorphic alleles is one of the important indicators of its functional relevance ^4,5^. We compare the similarity of different polymorphic representations of each orthologous loci to the four primate genomes (Chimpanzee panTro4, Orangutan ponAbe2, Gorilla gorGor3, Macaque rheMac3) to assign one of the representations with the ancestral state. We first construct the reference allele and the alternative alleles taking the flanking 500bp around the variant region into account. For a deletion compared with the reference, the reference allele is “left 500bp + deletion + right 500bp” and the alternative allele is “left 500bp + right 500bp”. For an insertion compared with the reference, the reference allele is “left 500bp + right 500bp” and the alternative allele is “left 500bp + insertion + right 500bp”. We align both the reference and the alternative alleles to the genome of four primates using last with the parameters used in Module a and categorize the variants as 0. “NONE” where both the reference and the alternative alleles cannot be aligned to any one of the primate genomes; 1. “NA” where both the reference and the alternative alleles can be aligned to one of the primate genomes but display less than 95% identity and 95% aligned ratio for all four primates 2. “Common” where both the reference and the alternative alleles display greater than 95% identity and aligned ratio for all four primates; 3. “Deletion” when the longer allele display greater than 95% identity and aligned ratio for any one of the primates and the shorter allele display less than 95% identity and aligned ratio for any one of the primates; 4. “Insertion” when the longer allele display greater than 95% identity and aligned ratio for any one of the primates and the shorter allele display less than 95% identity and aligned ratio for any one of the primates; 5.“Conflict” where the “Insertion” and “Deletion” judgment is different between different primates;

The strategy is similar to the ancestral annotation approach implemented in Breakseq ^6^ but we use last rather than blat which is more sensitive and efficient. The threshold of “95% identity” and “95% aligned ratio” is determined based on the distribution of the “NONE” alleles when applying the 99% and 99% thresholds (Data not shown).

### Module f. Annotation of Mechanism

We improve and implement the original breakSeqv1.3 approach [^21^](#_ENREF_21) to characterize the structural variants into different categories of mechanisms VNTR (Variable number tandem repeat), NAHR (Non-allelic homolog recombination), TEI (transposonable element insertions) and NHR (non-homologous recombination) ( **Figure1 SV Mechanism module** ).

| Mechanisms | Sequence features |
| --- | --- |
| CCC | Variation sequence that is exactly identical to the sequence with the same length on the 3’ of the breakpoint |
| VNTR | Variation sequence that are annotated as simple repeats, satellites and low complexity sequence by repeatmasker |
| TEI | Non-VNTR that are annotated as transposable elements by repeatmasker |
| NAHR | Variants where the two breakpoints share more than 85% identity |
| NHR (NHR-microhomogy) | Variants that are not annotated as the above and display micro-homologous sequence around the two breakpoints. |
| Unknown | Variants that do not display the above sequence features |

### Module g. Novel sequences analysis

The novel sequence analysis module identifies the sequences (>100bp by default) that are present in the de novo assemblies but cannot be aligned to the GRCh37 human genome sequence with greater than 95% identity and 95% aligned ratio (the length of the bases within the insertions that can be aligned to the reference divided by the length of the variants) and categorizes them into novel sequence insertions and nomadic novel sequence that cannot be localized in the human genome using the flanking sequences ( **Figure1 Novel Sequence module** ). By default, we realigned the sequences and obtained the novel sequences that were unambiguously aligned to the decoy sequence in 1KGP project, the de novo assemblies of an African, YH, NA12878, HuRef , the primate sequences and the other human genome sequences in the NT database using either last [^18^](#_ENREF_18) with the same parameters detailed in TableS1 or blastn [^20^](#_ENREF_20) .

### Evaluation of the false negative rate of AsmVar

We download the structural variation list from the 1KGP pilot project

from <ftp://ftp.ncbi.nlm.nih.gov/pub/dbVar/data/Homo_sapiens/by_study/estd59_1000_Genomes_Consortium_Pilot_Project>

and extract the 18932 structural variations that are validated in NA12878. We define those false negative calls as the structural variations that are present in the NA12878 dbVar calls but AsmVar fails to emit variation calls for the NA12878 individual.

### Sanger sequencing validation of the novel structural variants

We picked one trio 1298 from the GenomeDK consortium and validated a randomly selected set of variants present in the trio genomes using Sanger sequencing. The selected variants include 272 novel structural variants covering different size and mechanism spectrum. We design primers using an in-house pipeline integrating primer 3 and primer uniqueness checking. We sequenced the successfully amplified PCR amplicons the Sanger AB3730xI DNA Analyzer. We subsequently analysed chromatograms using PolyPhred 6.1849 to genotype SNVs and small indels. Hereafter all calls were manually inspected using Chromas 2.11.

#### qPCR validation of the novel sequence insertions(>=1000bp)

We design primers over the flanking regions of 18 novel sequences insertions that are greater than 1000bp. For a true novel sequence, we will observe bands with size more than 1000bp.

## Supplementary figures


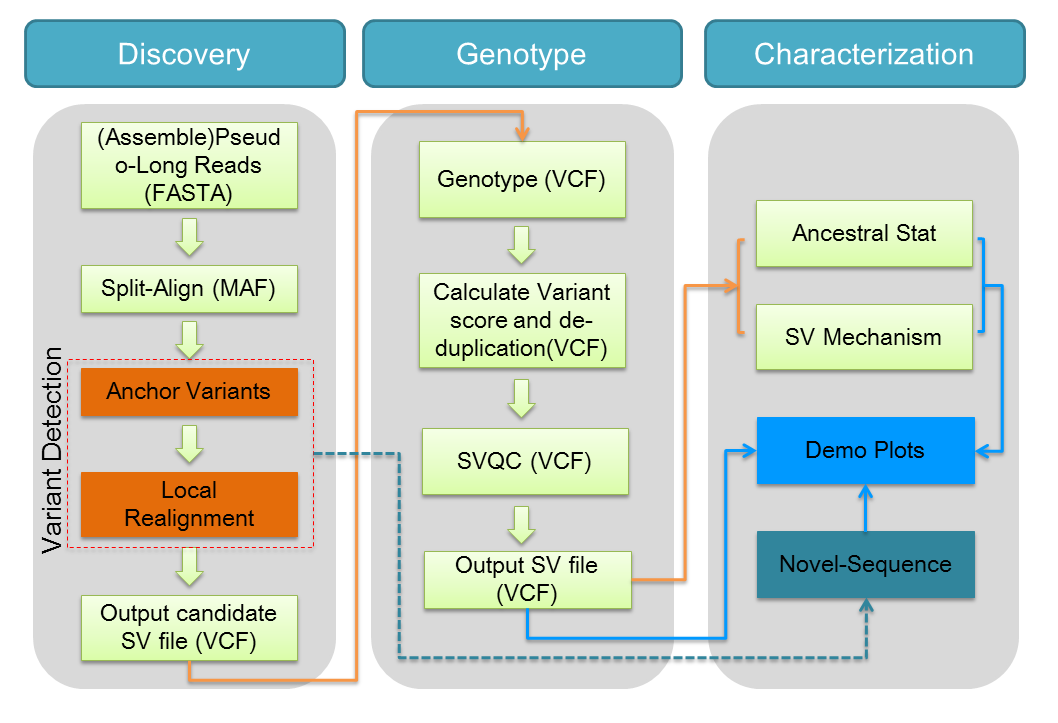


**FigureS1.** The AsmVar workflow


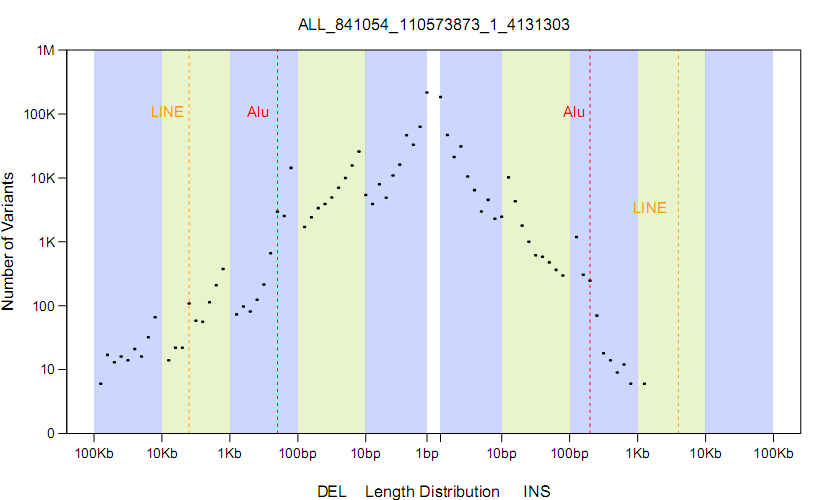


**FigureS2.** Size spectrum of the 841054 double hit events used as the positive training set in Module b in the 37 de novo assemblies investigation.


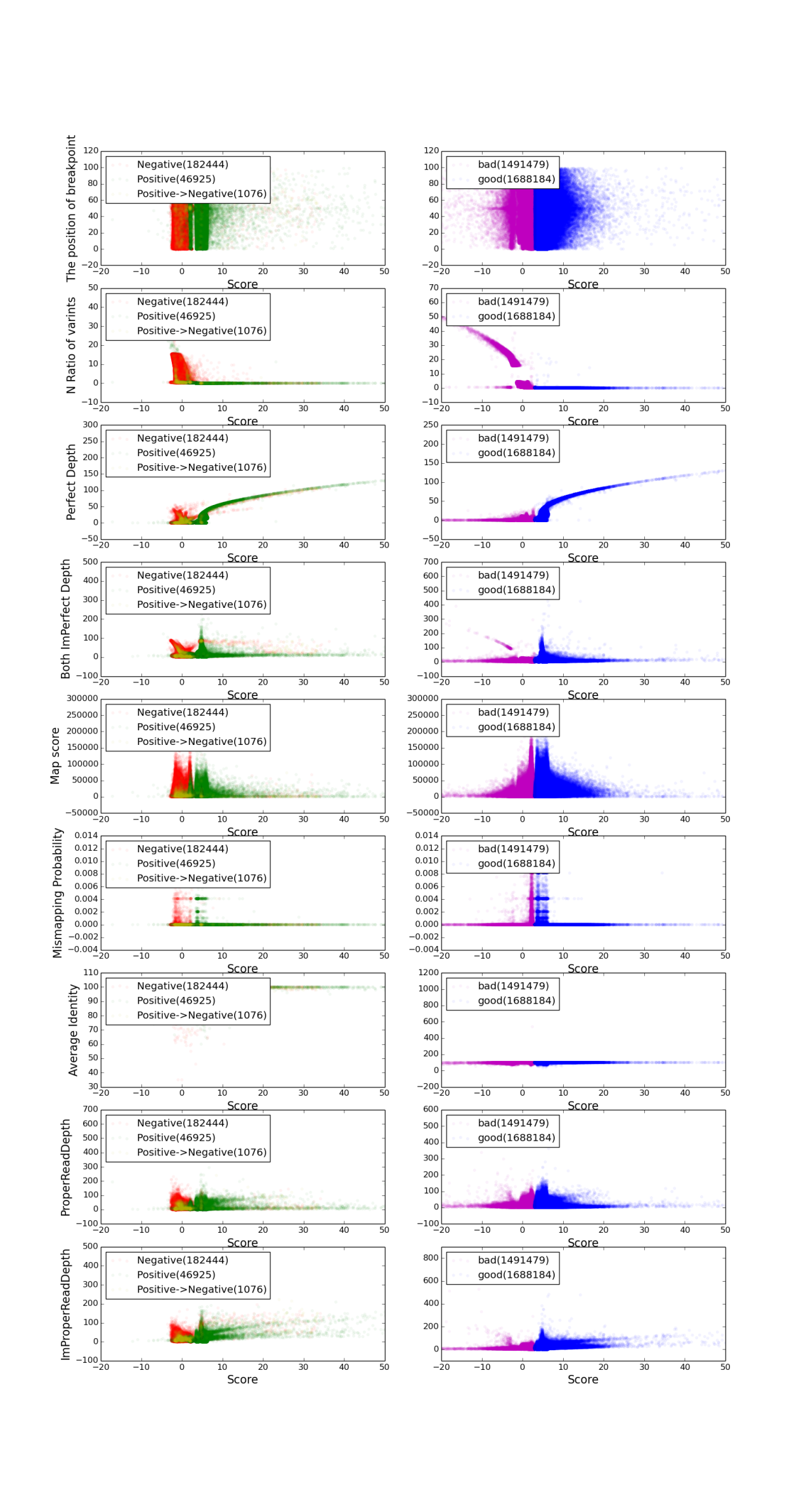


**FigureS3**. Variant quality score as a function of the distribution of the technical features in the AsmVar module c. Shown is the AsmVar’s application to current human genome de novo assemblies (N = 37).

The figure indicates that the classification of the variants based on the combined variant quality score is consistent with the expected distribution of different technical features. The positive variants are assigned with higher score compared with the negative variants. The most distinguishable features among the nine are the local N ratio of the variants (N ratio of variants) and the perfect read depth for the alternative allele present in the de novo assemblies (Perfect Depth), indicating that assembly quality is the main consideration for a complete profile of structural variants in human populations.

Left for training data set and Right for full data set:

Green/Blue: positive training sites/pass sites

Red/Rose red: negative training sites/false sites

Yellow: sites that swapped from positive to negative in the training model

x-axis- variant quality score

y-axis- raw measurement of a particular feature. The features are normalized in the final training.

Features illustrations:

(N ratio of variants) and the perfect read depth for the alternative allele present in the de novo assemblies (Perfect Depth)

1. The position of the breakpoint: the minimal difference between the coordinate of the breakpoint and the edge of the scaffold.
2. N ratio: Proportion of N bases in the de novo assembly within 200 base pairs around the breakpoints.
3. Perfect Depth: the depth of the reads that are uniquely and perfectly aligned to the alternative allele present in the de novo assembly.
4. Both Imperfect Depth: the depth of reads that are neither uniquely and perfectly aligned to the reference allele and the alternative alleles.
5. Map Score: alignment score of the alignment block that the variant exist (output by LAST).
6. Mismapping Probability: misalignment probability of the alignment block that the variant exist (output by LAST)
7. Average Identity: alignment identity of the flanking regions of the variant (output by AGE)
8. ProperReadDepth and ImProperReadDepth: depth of reads that are aligned to the de novo assembly around 50bp properly (see above “Alignment of short reads towards reference and the de novo assembly”)


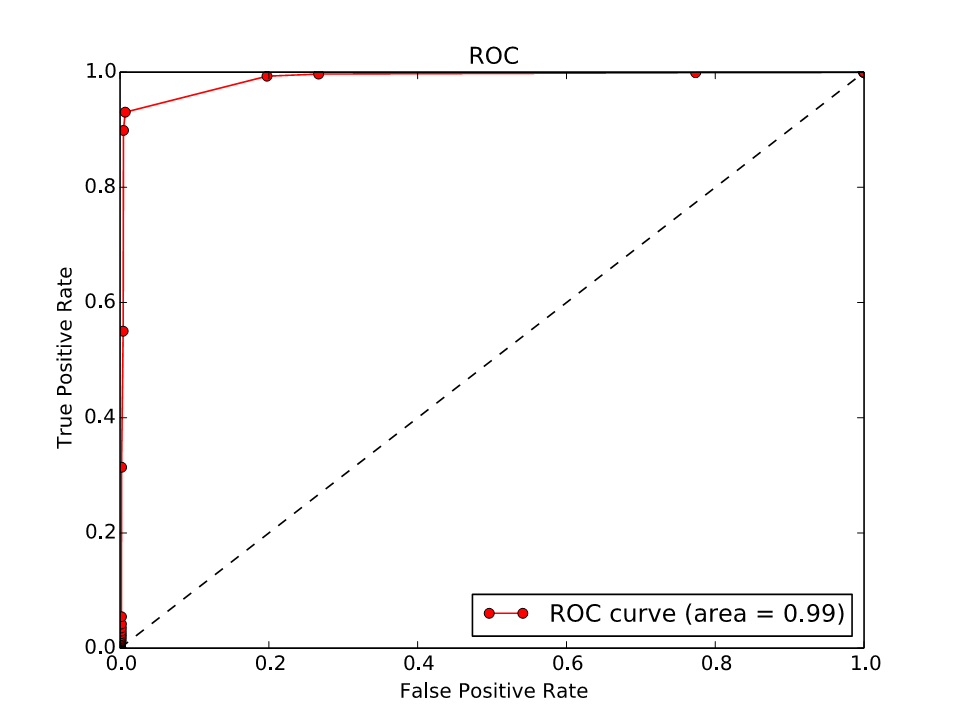


**FigureS4. ROC curve for variant quality threshold determination in the application**

When variant quality score is >=3, the True positive rate for the positive training set is ~93% and the false positive rate for the negative training set is ~0.7%.


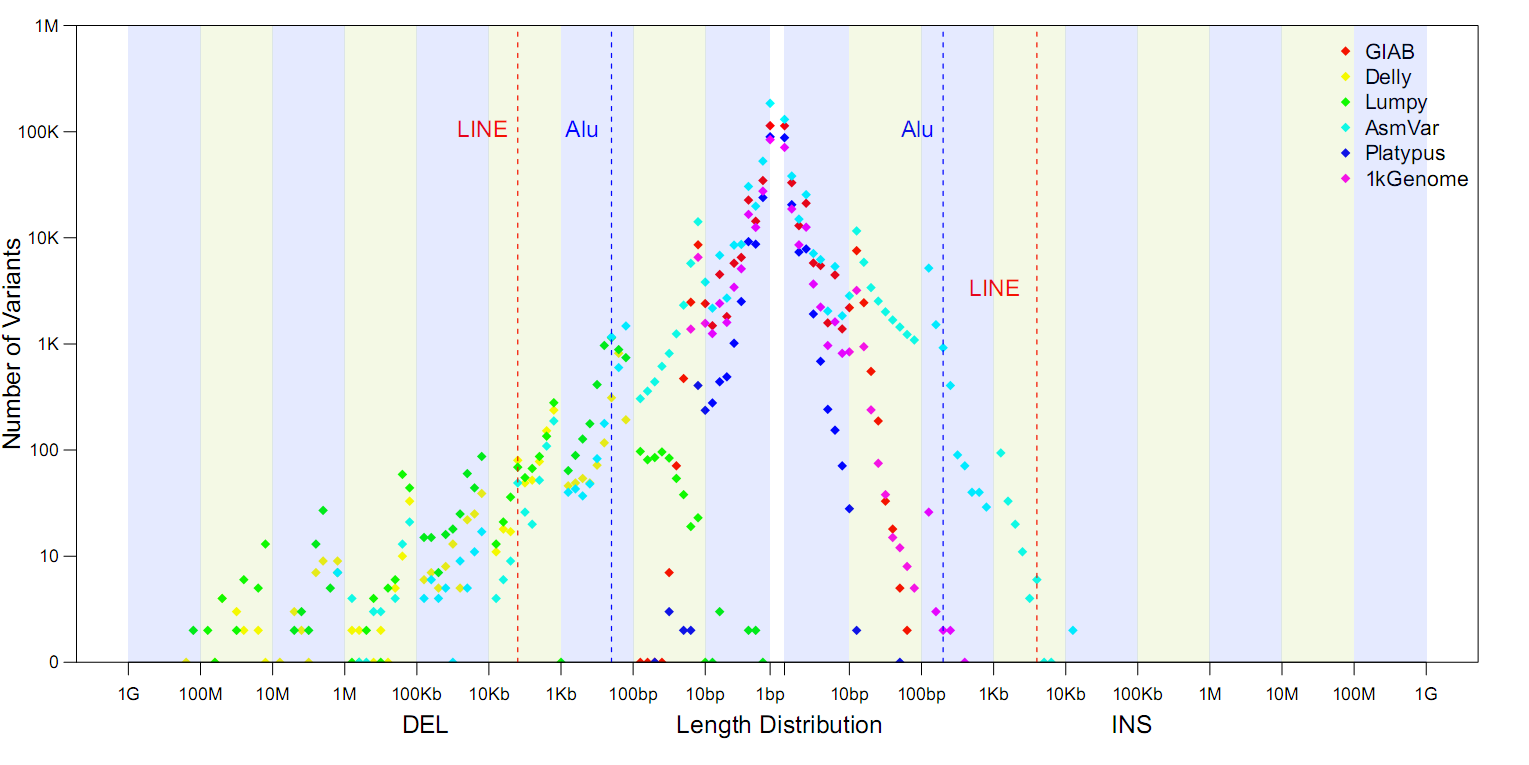


**FigureS5**. Size spectrum of the variation calls for NA12878 individual by AsmVar, Lumpy ^7^, Delly ^8^, Platypus^9^ and GATK ^3^ using the 40x high coverage data from 1KGP (<http://ftp.1000genomes.ebi.ac.uk/vol1/ftp/technical/working/20130103_high_cov_trio_bams/NA12878/alignment> ) and low coverage 1KGP PhaseIII release dataset ( <ftp://ftp.1000genomes.ebi.ac.uk/vol1/ftp/release/20130502> ). Lumpy and Delly vcf are downloaded from bcbio platform <https://s3.amazonaws.com/bcbio/sveval/NA12878-sv-validate.tar.gz> ; Platypus are run using the high coverage bam file from NA12878 in the 1000 genome consortium with default parameters; GATK results are downloaded from GenomeInABottleConsortium. “ftp://ftp-trace.ncbi.nih.gov/giab/ftp/data/NA12878/variant_calls/GIAB_integration/NIST_RTG_PlatGen_merged_highconfidence_v0.2_Allannotate.vcf.gz”.

As we observe from the size spectrum, GATK and Platypus calls restrict within 1bp to 20bp deletions and insertions. Lumpy and Delly display power mainly for deletions greater than 100bp. 1KGP variation discovery strategy by integrating information from multiple samples and difference softwares also display significant bias for variation. AsmVar shows power for deletions ranging from 1bp to 50kbp and insertions ranging from 1bp to 10kbp. The bias size spectrum suggests limitations of re-sequencing approach in identification of structural variation in human genomes.


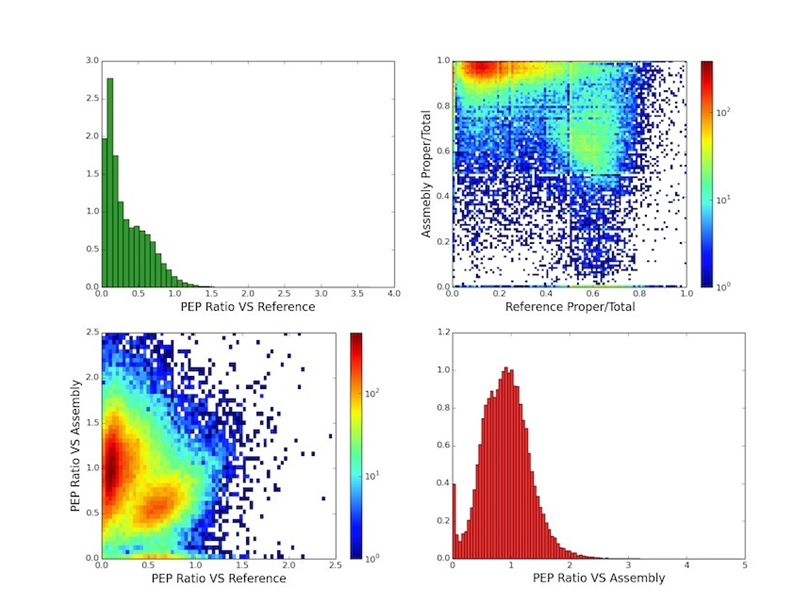


**FigureS6. Comparisons of the reference allele intensity and alternative allele intensity for the randomly selected novel structural variants identified in the application (N=6k)**

PEP ratio: Depth of the proper aligned reads within the variant loci normalized by that within the flanking the variant loci and variant size.

Proper/Total: Depth of the proper aligned reads within the variant loci normalized by the average sequencing read depth and variant size.

See “***Alignment of short reads towards reference and the de novo assembly***

” above for definition of proper aligned reads.

The plot suggests that evidence for the alternative allele present in the individual de novo assemblies is systematically higher than the allele present in the reference.

The three clusters are expected to be 1. Homozygous variants 2. Heterozygous structural variants and 3. Homozygous reference.


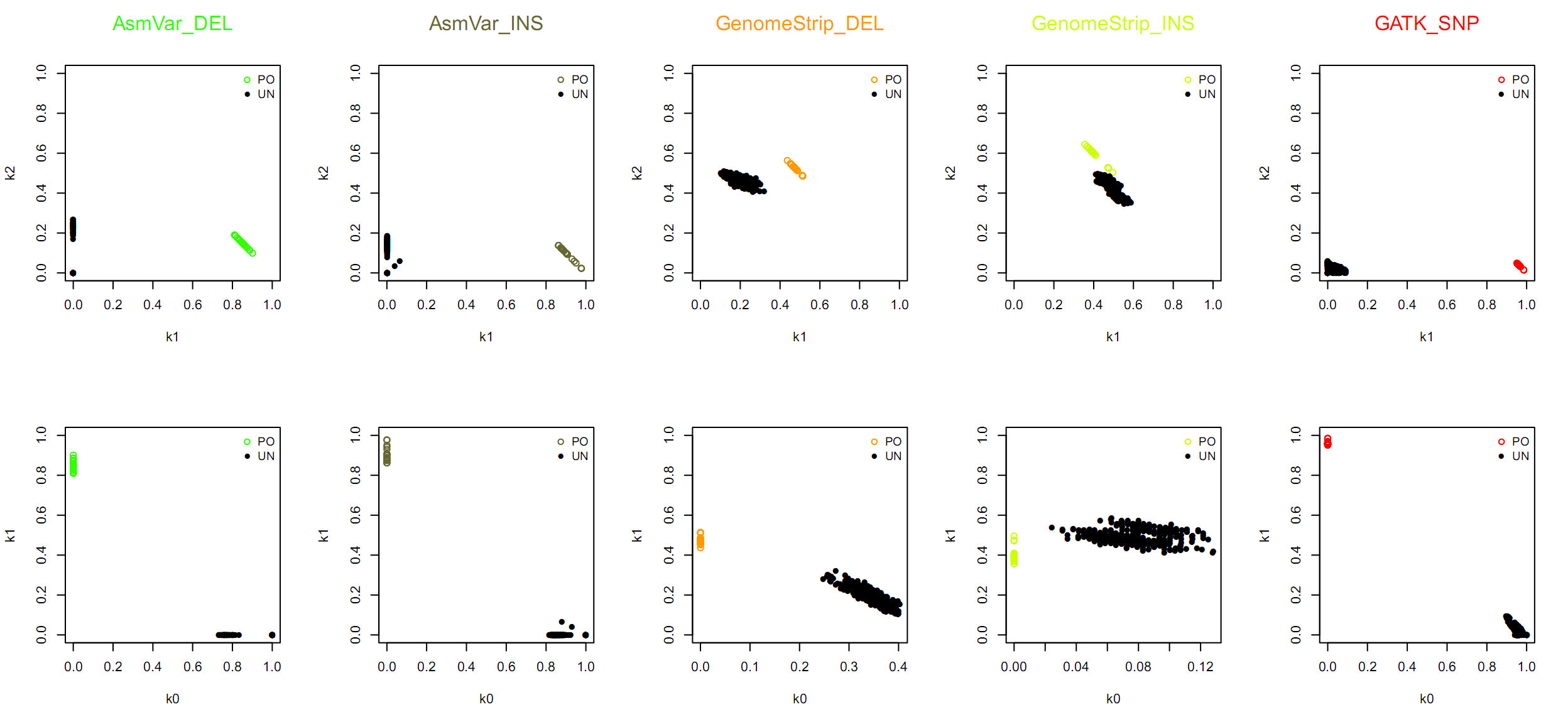


**FigureS7. Family relatedness using the 27684 deletions (>=50bp), 15065 insertions called from AsmVar (>=50bp); 10565 deletions and 3279 copy number variations from GenomeSTRIP**^10^ **and 8277766 SNPs from GATK for the 10 Danish Trio samples.**

Plink is used to estimate the family relatedness of the 10 Danish trios.

K0: IBD0

K1: IBD1

K2: IBD2

PO: parent-offspring

UN: unrelated individuals


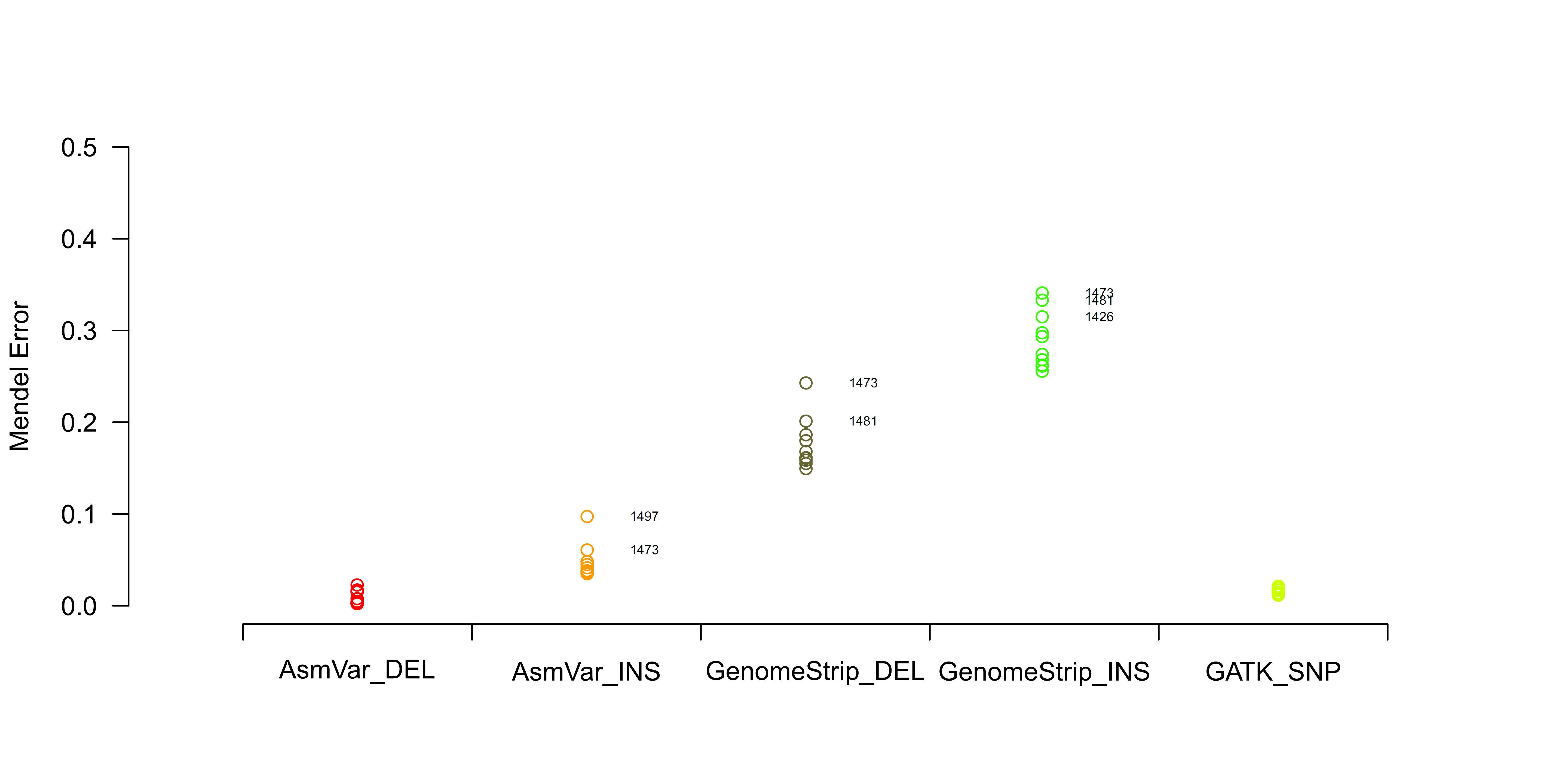


**FigureS8**. Shown is the Mendelian Error rate per trio for the deletions and insertions called by AsmVar (>=50bp) and the GenomeStrip (>=50bp), SNP called by GATK in10 Danish trios.


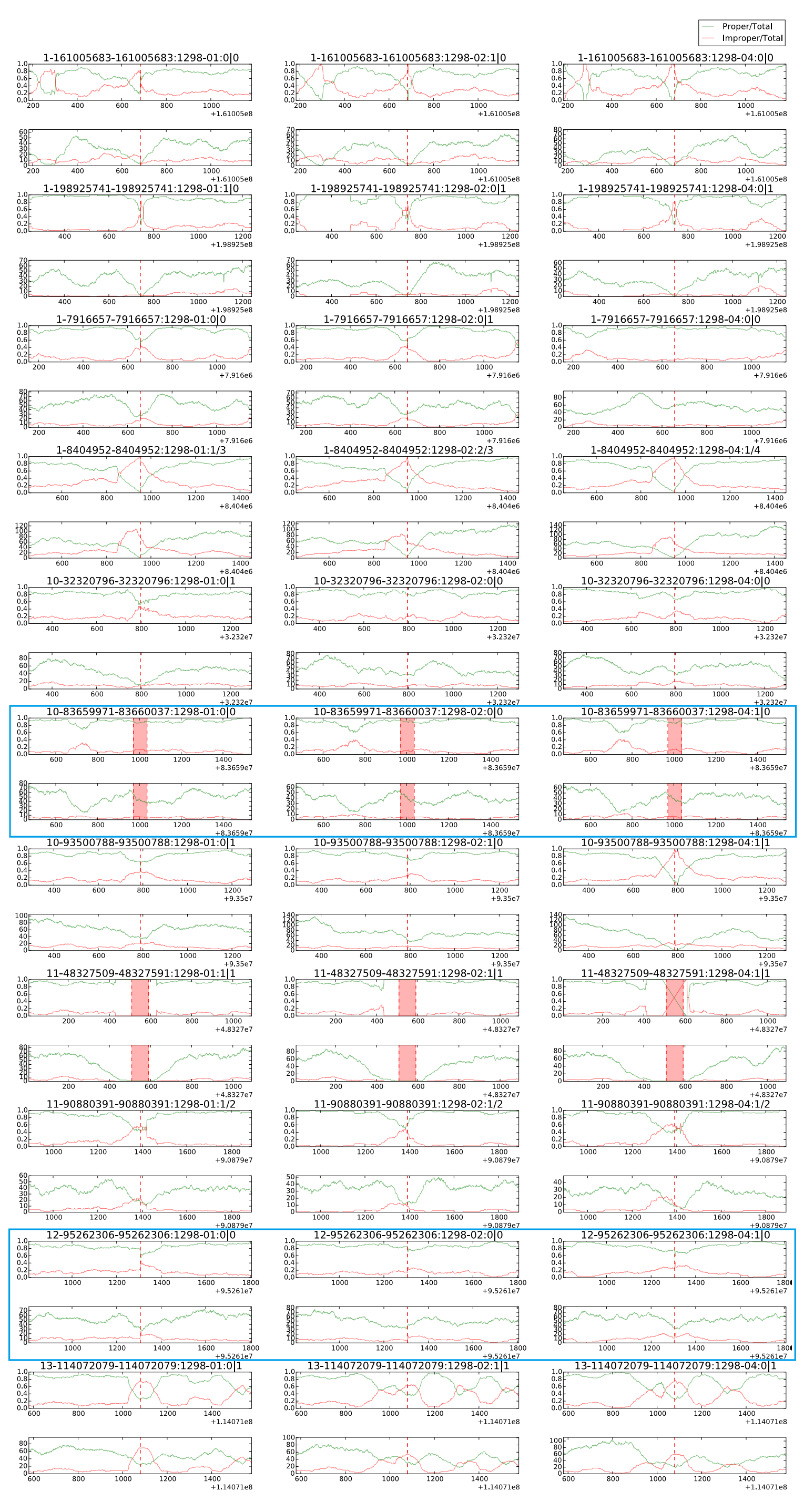


**FigureS9.** A snapshot of the read coverage around the11 out of the 46 and 158 loci failing experimental process. Each line represents one locus. For each locus, there are three individual profiles from the one trio 1298. For each individual, there are two sub-figures. The lower one indicates the proper and improper read coverage while the upper one describes the proper and improper read coverage normalized by the local depth. For one structural variation locus, we are expecting that we will observe lower proper read coverage and/or higher improper read coverage around the variation breakpoint compared to the flanking region.

The rest of the loci have been peer-reviewed and are available upon request. We didn’t include them in this additional file due to the solution limitations of figure pasted on the word document.


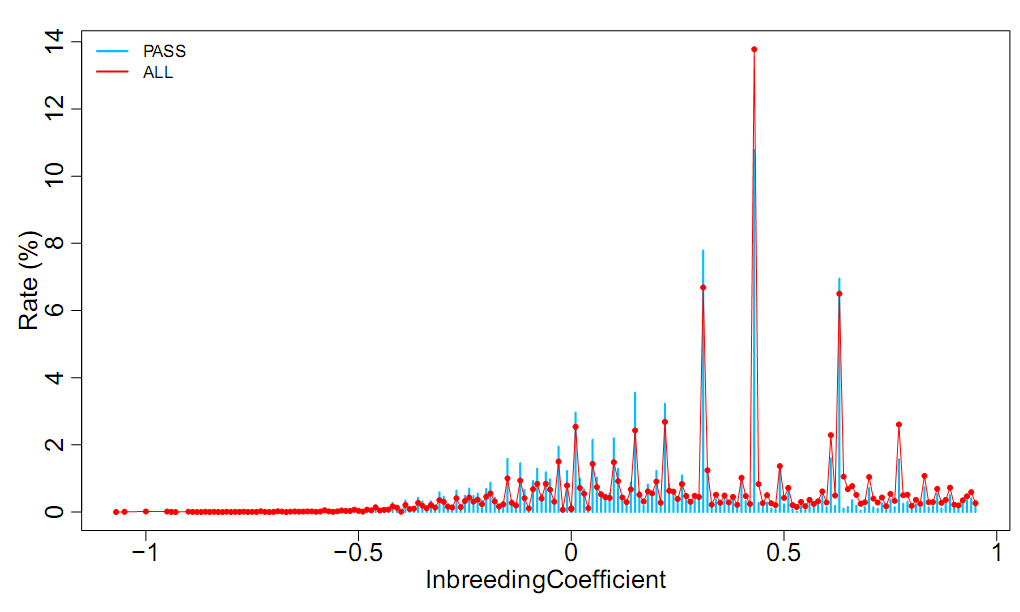


**Figure S10**. Distribution of the inbreeding coefficient. This figure is used by the users to determine the inbreeding coefficient threshold for posterior filtration of the variants.

## Supplementary tables

**TableS1: Information of the 37 Human genome de novo assemblies that are used in this analysis**

**TableS2. Memory and CPU time of AsmVar for the 37 de novo assembly investigation**

**TableS3. Assessment of AsmVar false negative rate by comparison of NA12878 validated structural variants**

**TableS4. False positive rate of AsmVar evaluated by Sanger sequencing validation**

**TableS5. qPCR to validate 18 novel sequences > 1 kbp in trio 1298**

## Supplementary Reference

1. Kiełbasa, S. M., Wan, R., Sato, K., Horton, P. & Frith, M. C. Adaptive seeds tame genomic sequence comparison. *Genome Res.* **21,** 487–93 (2011).

2. Abyzov, A. & Gerstein, M. AGE: defining breakpoints of genomic structural variants at single-nucleotide resolution, through optimal alignments with gap excision. *Bioinformatics* **27,** 595–603 (2011).

3. DePristo, M. a *et al.* A framework for variation discovery and genotyping using next-generation DNA sequencing data. *Nat. Genet.* **43,** 491–8 (2011).

4. MacArthur, D. G. *et al.* A systematic survey of loss-of-function variants in human protein-coding genes. *Science* **335,** 823–8 (2012).

5. Kiezun, A. *et al.* Deleterious Alleles in the Human Genome Are on Average Younger Than Neutral Alleles of the Same Frequency. *PLoS Genet.* **9,** 1–12 (2013).

6. Lam, H. Y. K. *et al.* Nucleotide-resolution analysis of structural variants using BreakSeq and a breakpoint library. *Nat. Biotechnol.* **28,** 47–55 (2010).

7. Layer, R. M., Chiang, C., Quinlan, A. R. & Hall, I. M. LUMPY: a probabilistic framework for structural variant discovery. *Genome Biol.* **15,** R84 (2014).

8. Rausch, T. *et al.* DELLY: structural variant discovery by integrated paired-end and split-read analysis. *Bioinformatics* **28,** i333–i339 (2012).

9. Rimmer, A. *et al.* Integrating mapping-, assembly- and haplotype-based approaches for calling variants in clinical sequencing applications. *Nat. Genet.* 1–90 (2014). doi:10.1038/ng.3036

10. Handsaker, R. E., Korn, J. M., Nemesh, J. & McCarroll, S. a. Discovery and genotyping of genome structural polymorphism by sequencing on a population scale. *Nat. Genet.* **43,** 269–76 (2011).
